# Recent Trends in Nanomaterial-Based Biosensors for Point-of-Care Testing

**DOI:** 10.3389/fchem.2020.586702

**Published:** 2020-10-16

**Authors:** Xu Wang, Feng Li, Yirong Guo

**Affiliations:** ^1^Wyss Institute for Biologically Inspired Engineering, Harvard University, Boston, MA, United States; ^2^College of Chemistry, Sichuan University, Chengdu, China; ^3^Department of Chemistry, Brock University, St. Catharines, ON, Canada; ^4^Institute of Pesticide and Environmental Toxicology, Zhejiang University, Hangzhou, China

**Keywords:** biosensors, POCT (point-of-care testing), nanomaterials, diagnostics, microfluidics

## Abstract

In recent years, nanomaterials of different shape, size, and composition have been prepared and characterized, such as gold and silver nanoparticles, quantum dots, mesoporous silica nanoparticles, carbon nanomaterials, and hybrid nanocomposites. Because of their unique physical and chemical properties, these nanomaterials are increasingly used in point-of-care testing (POCT) to improve analytical performance and simplify detection process. They are used either as carriers for immobilizing biorecognition elements, or as labels for signal generation, transduction and amplification. In this commentary, we highlight recent POCT technologies that employ nanotechnology for the analysis of disease biomarkers, including small-molecule metabolites, enzymes, proteins, nucleic acids, cancer cells, and pathogens. Recent advances in lateral flow tests, printable electrochemical biosensors, and microfluidics-based devices are summarized. Existing challenges and future directions are also discussed.

## Introduction

Point-of-care testing (POCT) refers to medical diagnostic tests performed near the place and time of patient care. This is in contrast to the traditional model in which testing is completely or mostly conducted in central laboratories and it takes hours or days to obtain results (Syedmoradi et al., 2017). Biosensors are widely used for POCT. A biosensor is an analytical device that can detect target analytes by converting the biological reaction into a measurable signal through the combination of biological recognition components with physical transducers (Su et al., 2017). The most commonly used readout modalities include optical, electrochemical, piezoelectric, magnetic, thermal, and colorimetric detection (Pashchenko et al., 2018). Many reagents can be used as the biological recognition component, such as nucleic acids, antibodies, enzymes, ligands and receptors, and molecular imprinted polymers (Lee et al., 2012). Biosensors have been utilized for the detection of various analytes such as proteins, nucleic acids, small molecules, enzymes, cells, and microorganisms. Biosensors are widely used in food industry, diagnosis, imaging, DNA sequencing, and biodefense. In this paper, we will focus on their applications in medical diagnosis.

**Figure 1:**
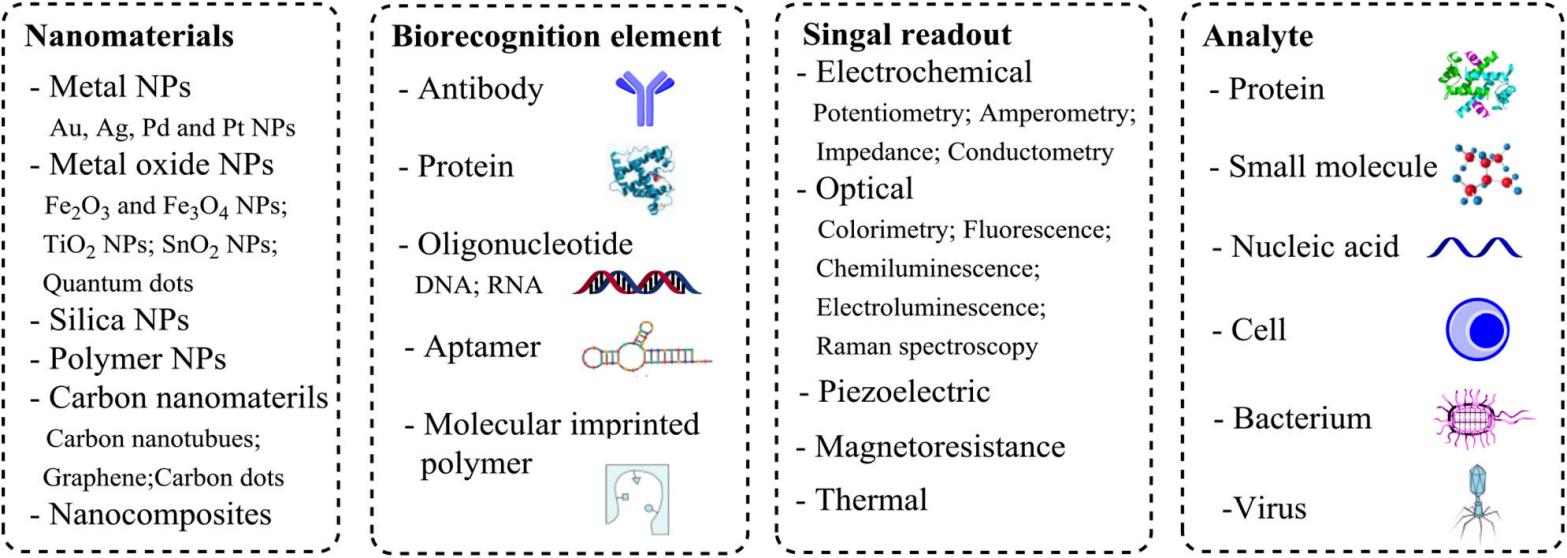
Components and readout formats associated with nanomaterial-based biosensors.

Recently, nanomaterials of different shape, size and composition have been prepared and characterized, such as gold and silver nanoparticles, quantum dots, mesoporous silica nanoparticles, carbon nanomaterials including carbon nanotubes (CNTs) and graphene, and hybrid nanocomposites (Reddy et al., 2012; Saha et al., 2012; Yin and Talapin, 2013; Hong et al., 2015). Compared with their bulk counterparts, nanomaterials have some unique physical and chemical properties, such as large surface area, excellent biocompatibility, and specific catalytic activity, which make nanomaterials an excellent candidate for manufacturing detection probes (Colombo et al., 2012; Jans and Huo, 2012). Nanomaterials are increasingly used in biosensors for biomarker detection (Holzinger et al., 2014; Quesada-González and Merkoçi, 2018). Because of their large surface area, nanomaterials are often used as carriers for immobilizing biorecognition elements. In addition, they are utilized as optical, electrochemical, or magnetic labels for signal generation, transduction and amplification (Wang et al., 2016). We will discuss applications of nanomaterials in biosensors for POCT and how they can improve the analytical performance (Figure 1).

## Current Poct Technologies

Pregnancy test strips and blood glucose meters are the two most classic and commonly used POCT devices. In recent years, some new detection technologies have been used in POCT, such as magnetoresistive response, time-resolved fluorescence and upconversion luminescence. By combining these detection methods with nanotechnology, a variety of new devices have been developed. In this section, recent advances in POCT technologies are summarized and discussed.

### Lateral Flow Strips

At the beginning, lateral flow tests are mainly used for detecting human chorionic gonadotropin (hCG) to determine whether or not a woman is pregnant. Now the same assay format has been adapted for detecting many analytes such as cardiovascular biomarkers and infectious pathogens. Different types of samples such as blood, urine, saliva, sweat, and other body fluids can be analyzed by lateral flow assays for disease diagnosis (Quesada-González and Merkoçi, 2015).

**Figure 2:**
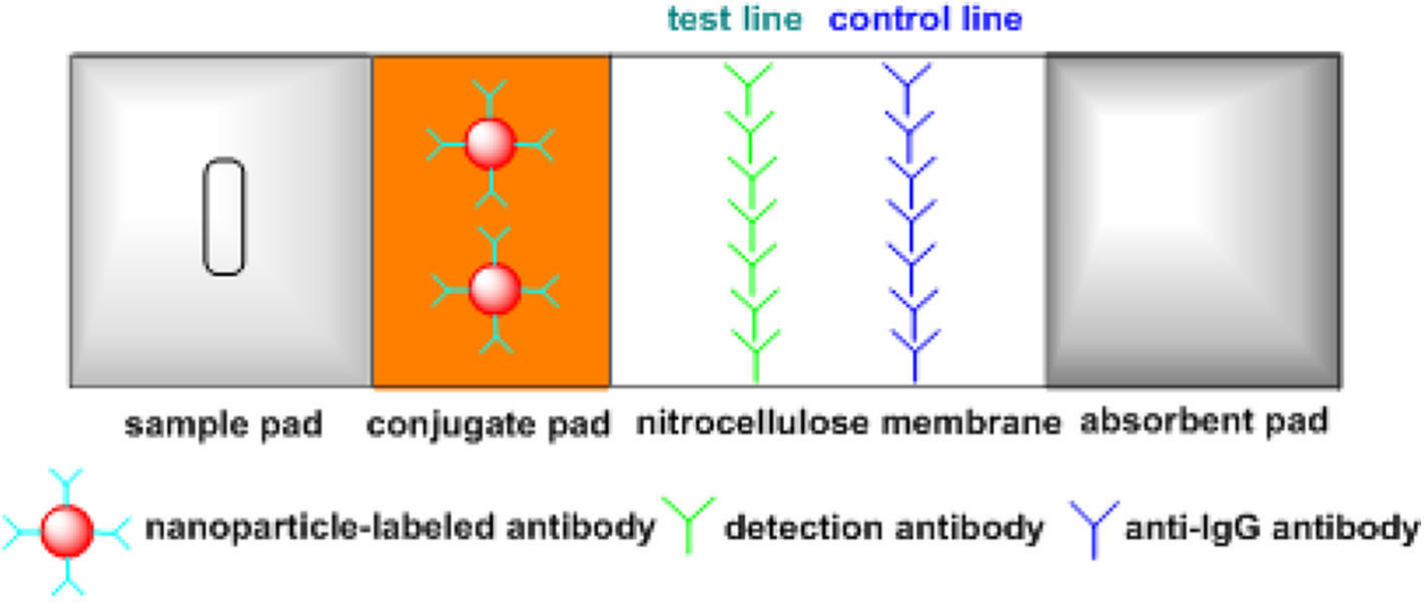
Schematic of a lateral flow device for detecting proteins.

Figure 2 shows the schematic illustration of a lateral flow strip for detecting proteins. It contains four main parts, i.e., sample pad, conjugate pad, nitrocellulose membrane, and absorbent pad. Specifically, the sample pad ensures the contact of liquid sample with the strip. The conjugate pad is preloaded with nanogold-labeled antibodies for signal generation. Two lines are drawn on the nitrocellulose membrane, i.e., the control and test lines. The test line is used for the detection of target analyte and the control line is to confirm the testing is working properly. Under the effect of capillary force, the liquid sample moves along the strip and nanogold-labeled antibodies are transferred to the nitrocellulose membrane. In the presence of target analyte, the target molecules are captured by nanogold-labeled antibodies and then bind with detection antibodies on the test line. Meanwhile, the nanogold-labeled antibodies are captured by anti-IgG antibodies on the control line, so both lines turn red. In the absence of target analyte, only one red line (control line) is observed. In addition, a competitive immunoassay format can be used for detecting small molecules such as progesterone, testosterone and estradiol. Gold nanoparticles (AuNPs) are coated with detection antibodies, and the target small molecules in liquid sample compete with immobilized analyte-protein conjugates on the test line for binding to AuNP-labeled detection antibodies. In the absence of analyte, the test line is red, while the color of test line becomes lighter as the analyte concentration increases. For example, Yang et al. developed a competitive lateral flow test for rapid detection of estradiol within the range of 37.14–1484.65 pg/mL (Yang et al., 2015). Additionally, multiple test lines can be used for simultaneous detection of different targets (Song et al., 2014). For instance, Tsai et al. developed a multiplex and sensitive lateral flow assay for simultaneous detection of alpha-defensin and C-reactive protein (Tsai et al., 2019).

The first generation of lateral flow tests use latex beads and AuNPs as the label. Because the label has a color, test result can be directly determined by naked eyes, but this is only used for qualitative analysis. To make quantitative measurements, strip readers are used to detect scattering light from AuNPs. However, the sensitivity of the first generation tests is relatively low, which can only be used for detecting analytes with relatively high concentrations. To improve the analytical performance, nanomaterials such as quantum dots, upconversion nanoparticles (UCNPs) and dye-loaded fluorescent nanoparticles are used as labels (Gong et al., 2017, 2019; Wang et al., 2019) Strip readers containing specific optics are used to measure the optical signal. For example, Tao's group developed quantum dot-based lateral flow immunoassays for detecting serum-specific IgE against *Dermatophagoides pteronyssinus* (Der-p) and *Dermatophagoides farinae* (Der-f) (Liang et al., 2020), which can detect serum-specific levels of IgE against Der-p and Der-f as low as 0.093 and 0.087 IU/mL, respectively. Jin et al. developed quantitative lateral flow immunoassays using highly doped UCNPs for the detection of prostate specific antigen (PSA) and ephrin type-A receptor 2 with detection limits of 89 and 400 pg/mL, respectively (He et al., 2018). Lateral flow tests are simple, economic and generally show results in around 5–20 min, which are widely used in medical diagnosis. Besides proteins and small molecules, lateral flow tests can be used for detecting other analytes such as lysophospholipids, pathogens and nucleic acids (Zhu et al., 2016; Takalkar et al., 2017; Tominaga, 2019). For example, Lu et al. developed a lateral flow test for detecting plasma lysophosphatidic acid using polydiacetylenes-based probe (Wang et al., 2017). Although very simple and fast, lateral flow tests usually have low sensitivity and poor reproducibility, because the reaction time on nitrocellulose membrane is too short and there is no wash step to reduce non-specific signals. In recent years, lateral flow strips have been used as the readout device and combined with some sensitive technologies such as loop-mediated isothermal amplification (LAMP), polymerase chain reaction (PCR), and CRISPR to construct other POCT detection platforms (Du et al., 2017; Gootenberg et al., 2018; Mauk et al., 2018). For instance, Broughton et al. developed a CRISPR–Cas12-based lateral flow assay for detection of betacoronavirus severe acute respiratory syndrome (SARS)-CoV-2 from extracted patient sample RNA within 40 min (Broughton et al., 2020). A PCR-based lateral flow assay was established for the detection of canine parvovirus 2 with detection limit of 30 copies/μL (Zhuang et al., 2019). In addition, Li et al. developed a LAMP-based lateral flow biosensor for rapid detection of *Brucella* spp. with detection limit of 100 fg per reaction (Li et al., 2019).

### Printable Electrochemical Biosensors

**Figure 3:**
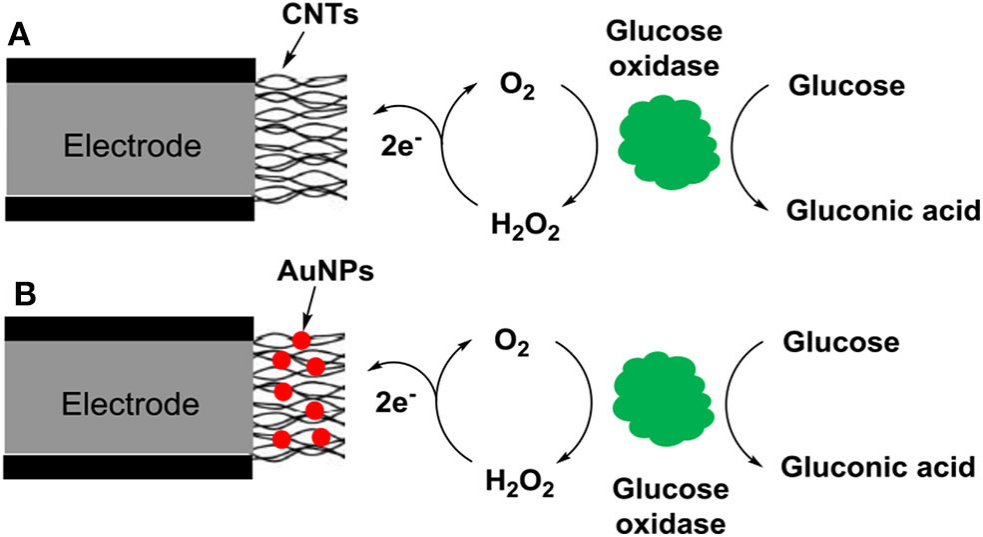
Schematic of nanomaterial-based electrochemical biosensors for detecting glucose: **(A)** single type of nanomaterials (CNTs) and **(B)** hybrid nanocomposites consisting of multiple nanomaterials (CNTs and AuNPs) are used.

Home-use blood glucose meters are the most well-known electrochemical biosensors for POCT (Cash and Clark, 2010). The electrochemical biosensor usually contains three electrodes, i.e., the working and counter electrodes, as well as the reference electrode. The main electrode is the working electrode where electrochemical reactions occur, while the counter and reference electrodes are utilized to complete the electronic circuit (Syedmoradi et al., 2017). Screen-printing technology is usually applied for large-scale production of low-cost electrochemical biosensors. Nanomaterials are widely utilized in the fabrication of electrochemical biosensors to improve the analytical performance, because they can increase surface area, improve electrocatalytic activity, and accelerate electron transfer to electrodes. Either single type of nanomaterials, such as CNTs, Fe_3_O_4_ particles and AuNPs, or hybrid nanocomposites consisting of multiple nanomaterials such as a combination of CNTs and AuNPs can be used (Figure 3) (Su et al., 2017). Printable electrochemical biosensors have been utilized for detecting a variety of analytes such as proteins, enzymes, and nucleic acids (Yamanaka et al., 2016). For example, Omidi's group developed a sensitive electrochemical biosensor using graphene oxide–gold nanostructures for detecting PSA with detection limits of 0.2 and 0.07 ng/mL for total and free PSA antigen, respectively (Akbari jonous et al., 2019). Ilkhani et al. developed an electrochemical aptamer/antibody based immunosensor using AuNPs as the signaling probe for detecting EGFR with detection limit of 50 pg/mL (Ilkhani et al., 2015). A printable voltammetric genosensor based on a nanocomposite of nano-polyaniline and graphene oxide (GO)-ceria nanoparticles was developed for detecting mutation in a particular sequence of the adenomatous polyposis coli gene (Eskandari and Faridbod, 2018). The analysis is usually rapid, low cost, and highly sensitive. And it is relatively easy to miniaturize the device, which is especially important for the utility in POCT. However, the interference of complex sample matrices may limit the practical application of these biosensors.

### Devices With Precise Fluid Control

Precise fluid control is usually required to make the assay more accurate and improve reproducibility. This can be achieved by incorporating a series of pumps and valves into the test instrument. For POCT applications, the size and complexity of the instrument need be considered to ensure that it can be operated by personnel without laboratory training (St John and Price, 2014). In recent years, with the rapid development of microfluidic technologies, pumps and valves can be incorporated into a small microfluidic chip and manipulated by a matching chip reader (Sia and Kricka, 2008; Pandey et al., 2018). The chip reader is usually portable, so it can be used at the bedside or in the clinic, and operated by patients themselves or healthcare professionals. For instance, Jiang's group developed an automated microfluidic chemiluminescence immunoassay for quantitative detection of protein biomarkers (Hu et al., 2017). McDevitt's group developed a portable microfluidic device for quantification of protein biomarkers using plastic disposable cartridges (McRae et al., 2015). In addition, many paper-based microfluidic devices have been developed in which no pump is needed for fluidic control (Akyazi et al., 2018). For example, Ansari et al. developed a portable microfluidic paper-based analytical device for on-site blood detection and blood typing (Ansari et al., 2020). Rosenfeld et al. developed a paper-based microfluidic device for amplification-free detection of DNA using electroosmotically balanced isotachophoresis (Rosenfeld and Bercovici, 2018). A variety of nanomaterials, such as quantum dots, UCNPs, CNTs, graphene oxide, and magnetic nanoparticles, have been used in these microfluidic assays (Mou and Jiang, 2017; Zhang and Misra, 2019). Different readout modalities, such as colorimetry, fluorescence, chemiluminescence, upconversion luminescence, and magnetoresistive response, can be used to measure the signal (Zhu and Fang, 2013). Microfluidic assays have been used for detecting various analytes including proteins, enzymes, nucleic acids, cancer cells, bacteria and viruses (McCalla and Tripathi, 2011; Sackmann et al., 2014). For example, Chand et al. developed a microfluidic platform integrated with graphene-gold nanocomposite aptasensor for detecting norovirus with a detection limit of 100 pM (Chand and Neethirajan, 2017). A smartphone-based point-of-care platform for detecting avian influenza virus was developed by Xia et al. using AuNP-based silver enhancement (Xia et al., 2019). Gao et al. developed a microfluidic point-of-care device for simultaneous detection of multiple genetic targets using quantum dot-barcoded microbeads (Gao et al., 2013). Compared with conventional lateral flow tests and electrochemical biosensors, microfluidic assays have many advantages such as high sensitivity and good reproducibility. But usually the fabrication of microfluidic cartridges is complicated and costly.

## Remaining Challenges and Future Directions

Although nanomaterials have been widely used in biosensors and dramatically improve the analytical performance, there are still some limitations. One key challenge is the sensitivity. Many potentially useful targets, such as cancer and cardiovascular biomarkers, exist in biological samples with concentrations far below the detection limit of current methods. Thus, it is essential to develop more sensitive techniques. Furthermore, many brain-derived biomarkers for neurodegenerative diseases, such as neurofilament light and amyloid beta peptide 42, are present in cerebrospinal fluid (CSF) with relatively high concentrations, while their concentrations in blood are very low (Wu et al., 2019). A sample of CSF is usually taken *via* lumbar puncture, which is highly invasive. Improved sensitivity can allow the detection of these biomarkers directly from the blood via minimally invasive sampling. In addition, detection of biomarkers in saliva or urine is another promising direction. Saliva and urine can be easily collected and analyzed, which can potentially promote early diagnosis and long-term monitoring of many diseases, e.g., infectious diseases, neurodegenerative diseases, and cancers. Because the biomarkers are highly diluted in saliva and urine, higher analytical sensitivity is usually required. Nanomaterials can be used to improve detection sensitivity due to their unique properties. For instance, nanomaterials have a large surface area so they can be used to load a high number of reporter molecules like enzymes and fluorophores for signal amplification. Furthermore, some ultrasensitive detection techniques such as digital PCR and single molecule analysis can be combined with nanotechnology to further improve the sensitivity (Cohen and Walt, 2017; Sreejith et al., 2018). Moreover, some nanomaterials such as Fe_3_O_4_, palladium and platinum nanoparticles have very high catalytic activity so they can be directly used as reporters for signal generation (Liu et al., 2019).

Another challenge is to make multiplexed measurements. Currently most assays only detect one analyte at a time. Biological samples usually contain a large variety of molecules such as proteins, nucleic acids, and small-molecule metabolites. These substances regulate biological processes in a collaborative manner (Liu et al., 2018). Thus, it is significant to simultaneously analyze multiple analytes. Multiplex assay can obtain more information and improve detection efficiency. This is especially useful for situations in which the sample volume is limited. For example, the collection of CSF is highly invasive, so the sample is precious and it is highly desirable to simultaneously detect multiple biomarkers in CSF. Nanomaterials with different size and shape or fluorescent nanoparticles with different emission wavelength can be used to make multiplexed detection.

Although many nanomaterial-based biosensors are reported every year, only a very few of them are successfully used in real clinical trials. The main challenges of nanomaterial-based biosensors are reproducibility and robustness. In practice nanomaterials with complex structures are often used in biosensors to improve detection sensitivity and specificity. However, the morphology of these nanomaterials may vary from batch to batch, leading to a low reproducibility. In addition, non-specific interaction is an inevitable issue in most assays. Thus, more efforts are needed to better understand the nanomanufacturing process and control particle aggregation and surface interactions. For clinical applications, strict quality control is required to ensure that the measurements have good reproducibility and accuracy.

## Concluding Remarks

In this perspective paper, we described some recent advancement in nanomaterial-based biosensors for point-of-care diagnostics, mainly including lateral flow assays, printable electrochemical biosensors, and microfluidic devices. Undoubtedly, the rapid development of nanotechnology has promoted significant progress in POCT analysis. The problems of conventional biosensors, such as low specificity and sensitivity, have been partially resolved by using nanomaterials. Nanomaterial-based biosensors have been increasingly utilized in clinical diagnosis. However, there are still some issues that limit large-scale applications of these biosensors and devices in clinical, such as relative high cost, lack of multiplexibility, relatively low reproducibility, and moderate sensitivity and specificity. The future direction of nanomaterial-based biosensors is to miniaturize the instrument, reduce analysis time and cost, while improving the reproducibility, sensitivity, and detection efficiency. There is no doubt that in the near future more nanomaterial-based POCT devices will be developed and utilized in clinical trials, particularly in low-resource, laboratory-free settings. In addition, we believe nanomaterial-based biosensors will also attract more and more attention in other areas such as food safety and environmental analysis.

## Data Availability Statement

All datasets generated for this study are included in the article/supplementary material.

## Author Contributions

XW and YG wrote the manuscript. FL revised the manuscript. All authors contributed to the article and approved the submitted version.

## Conflict of Interest

The authors declare that the research was conducted in the absence of any commercial or financial relationships that could be construed as a potential conflict of interest.
